# High expression of IL6 and decrease in immune cells in COVID-19 patients combined with myocardial injury

**DOI:** 10.3389/fimmu.2023.1190644

**Published:** 2023-07-26

**Authors:** Tingting Chen, Haixin Chen, Ping Chen, Linchao Zhu, Wei Mao, Yimin Yao

**Affiliations:** ^1^ Medical Laboratory, The First Affiliated Hospital of Zhejiang Chinese Medical University (Zhejiang Provincial Hospital of Chinese Medicine), Hangzhou, China; ^2^ The First Clinical Medical College, Zhejiang Chinese Medical University, Hangzhou, China; ^3^ Department of Cardiology, Zhejiang Hospital (Affiliated Zhejiang Hospital Zhejiang University School of Medicine), Hangzhou, China; ^4^ Key Laboratory of Integrative Chinese and Western Medicine for the Diagnosis and Treatment of Circulatory Diseases of Zhejiang Province, Hangzhou, China

**Keywords:** interleukin signal pathway, lymphocyte subsets, coronavirus disease-2019, myocardial injury, interleukin 6, high-sensitivity troponin

## Abstract

**Purpose:**

Myocardial injury, as a serious complication of coronavirus disease-2019 (COVID-19), increases the occurrence of adverse outcomes. Identification of key regulatory molecules of myocardial injury may help formulate corresponding treatment strategies and improve the prognosis of COVID-19 patients.

**Methods:**

Gene Set Enrichment Analysis (GSEA) was conducted to identify co-regulatory pathways. Differentially expressed genes (DEGs) in GSE150392 and GSE169241 were screened and an intersection analysis with key genes of the co-regulatory pathway was conducted. A protein-protein interaction (PPI) network was constructed to screen for key regulatory genes. Preliminarily screened genes were verified using other datasets to identify genes with consistent expression. Based on the hierarchical cluster, we divided the patients from GSE177477 into high- and low-risk groups and compared the proportion of immune cells. A total of 267 COVID-19 patients from the Zhejiang Provincial Hospital of Chinese Medicine from December 26, 2022, to January 11, 2023, were enrolled to verify the bioinformatics results. Univariate and multivariate analyses were performed to analyze the risk factors for myocardial injury. According to high-sensitivity troponin (hsTnI) levels, patients with COVID-19 were divided into high- and low-sensitivity groups, and interleukin 6 (IL6) expression and lymphocyte subsets were compared. Patients were also divided into high and low groups according to the IL6 expression, and hsTnI levels were compared.

**Results:**

Interleukin signaling pathway and GPCR ligand binding were shown to be co-regulatory pathways in myocardial injury associated with COVID-19. According to the hierarchical cluster analysis of seven genes (IL6, NFKBIA, CSF1, CXCL1, IL1R1, SOCS3, and CASP1), patients with myocardial injury could be distinguished from those without myocardial injury. Age, IL6 levels, and hospital stay may be factors influencing myocardial injury caused by COVID-19. Compared with COVID-19 patients without myocardial injury, the levels of IL6 in patients with myocardial injury increased, while the number of CD4^+^ T cells, CD8^+^ T cells, B cells, and NK cells decreased (*P*<0.05). The hsTnI levels in COVID-19 patients with high IL6 levels were higher than those in patients with low IL6 (*P*<0.05).

**Conclusions:**

The COVID-19 patients with myocardial injury had elevated IL6 expression and decreased lymphocyte counts. IL6 may participate in myocardial injury through the interleukin signaling pathway.

## Introduction

The syndrome-coronavirus-2 (SARS-COV-2) has infected nearly 600 million people and caused 6.5 million deaths worldwide. Approximately 12–41% of coronavirus disease-2019 (COVID-19) patients have myocardial injury (1). Myocardial injury is an independent risk factor of mortality (2). Cardiomyocytes infected with SARS-COV-2 showed a strong defense response, including excessive immune inflammation, cell hypoxia, decreased functional contractility, and myocardial injury (3). Myocardial injury also inhibits cardiomyocyte regeneration. Hartmann et al. suggested that infective myocardial injury caused by COVID-19 was mainly related to local inflammation with interstitial edema based on the autopsy of dead COVID-19 patients (4). Different researchers have proposed mechanisms for myocardial injury caused by COVID-19. However, the detailed mechanism underlying the myocardial injury caused by SARS-COV-2 infection is unclear. Identifying the main pathogenic mechanisms and key regulatory molecules will help treat COVID-19 patients with myocardial injury and reduce mortality.

The biological characteristics of myocardial injury caused by COVID-19 include inflammatory activation and immune cell infiltration (5). SARS-COV-2 causes inflammation of the myocardial vascular wall leading to small artery occlusion (6, 7). Complement and MAPK activation is also involved in myocarditis caused by SARS-COV-2 (8). Xie determined that the central genes related to the myocardium infected with SARS-COV-2 were ISG15, MX1, IFIT1, IFIT2, RSAD2, OAS2, IFIT3, MX2, OAS1, OAS3, IFI6, and DDX58 (9). These mechanisms were based on myocardial tissue samples. We attempted to screen common biomarkers between blood and tissue samples to predict the possibility of myocardial injury in COVID-19 patients. Blood sample acquisition is less traumatic and is more suitable for clinical use.

In this study, we analyzed the gene expression profile of COVID-19 datasets and found that the interleukin signaling pathway was typically abnormal through Gene Set Enrichment Analysis (GSEA). The gene set of the interleukin signaling pathway obtained from the GSEA website was subjected to intersection with differentially expressed genes (DEGs) from the COVID-19 datasets to obtain overlapping genes. These overlapping genes were further analyzed in uninfected healthy individuals, asymptomatic COVID-19 patients, and symptomatic COVID-19 patients with myocardial injury, and seven potential key genes were identified. Based on the hierarchical cluster diagram constructed using these seven genes, 45 clinical samples from GSE177477 were divided into low- and high-risk groups. Immune cells analysis showed that the proportions of B, CD4^+^T, CD8^+^T, and NK cells in the high-risk group were significantly lower than those in the low-risk group (*P*<0.05). Through bioinformatics analysis, we concluded that the COVID-19 patients combined with myocardial injury have abnormal interleukin pathways and immune cell imbalances. Next, the levels of interleukin 6 (IL6) and lymphocyte subsets in COVID-19 patients from our hospital were analyzed to verify whether there were abnormalities in interleukin and immune cells in COVID-19 patients with myocardial injury.

## Materials and methods

### Data sources

RNA-seq data (GSE150392 (10) and GSE169241 (11)) and whole-blood sample gene expression profiles (GSE177477 (12)) were downloaded from the NCBI Gene Expression Omnibus (GEO) database. Six samples in GSE150392 were divided into the Cov and mock groups. Three samples from the CoV group were human-induced pluripotent stem cell-derived cardiomyocytes (hiPSC-CMs) pretreated with SARS-COV-2 for 72 h. Three samples from the mock group were hiPSC-CMs treated with simulants without SARS-COV-2. GSE169241 included 23 samples, of which 3 were heart tissues from autopsied COVID-19 patients and 5 were heart tissues from donors without COVID-19. GSE177477 contained 18 asymptomatic COVID-19 cases, 11 symptomatic COVID-19 cases, and 18 uninfected healthy individuals. Among the 11 symptomatic cases, the description of myocardial injury was lacking for two patients and we were unable to determine whether they had myocardial injury. Finally, nine patients with symptomatic COVID-19 with myocardial injury were included.

A total of 267 COVID-19 patients from the Zhejiang Provincial Hospital of Chinese Medicine from December 26, 2022, to January 11, 2023, were enrolled for further analysis, including 159 males and 108 females (**Table 1**). We retrospectively reviewed the patients’ electronic medical records and analyzed their laboratory indicators. Patients with increased high-sensitivity cardiac troponin (hsTnI) levels after SARS-COV-2 infection are considered to have COVID-19-related myocardial injury. The main diagnostic criteria for myocardial injury caused by COVID-19 were positive results of the COVID-19 pathogenic test (COVID-19 nucleic acid test or COVID-19 antigen test) combined with an increase in hsTnI levels exceeding the upper limit of 99% of the reference value range (13). Given the close correlation between IL6 expression and the severity of inflammation, when analyzing the correlation between IL6 and hsTnI levels, we included data from blood collected during the same period.

**Table 1:** The Clinical feature of the patients.

Characteristics	hsTnI-L	hsTnI-H	*P* value
n	181	86	
Sex, n (%)			0.123
Male	102 (56.4%)	57 (66.3%)	
Female	79 (43.6%)	29 (33.7%)	
Age, median (IQR)	79 (71, 85)	87 (82, 90)	< 0.001*
Hypertension, n (%)	111 (61.3%)	61 (70.9%)	0.126
Diabetic mellitus, n (%)	49 (27.1%)	19 (22.1%)	0.383
Coronary atherosclerotic heart disease, n (%)	13 (7.2%)	12 (14%)	0.076
BMI (g/m2), median (IQR)	23.58 (21.22, 25.96)	19.98 (16.52, 24.66)	0.014*
Fasting Blood glucose (mmol/L), median (IQR)	6.98 (5.93, 9.10)	7.52 (6.00, 9.15)	0.175
Hemoglobin A1c (%), median (IQR)	6.35 (5.70, 7.18)	6.25 (5.75, 7.13)	0.672
Triglycerides (mmol/L), median (IQR)	1.12 (0.90, 1.55)	1.06 (0.76, 1.62)	0.284
Total cholesterol (mmol/L), mean ± SD	3.86 ± 1.00	3.34 ± 1.13	< 0.001*
IL6 (pg/mL), median (IQR)	10.14 (4.62, 30.84)	39.57 (13.75, 114.16)	< 0.001*
Hospital stay (days), median (IQR)	11.00 (8.00, 16.00)	13.50 (8.25, 21.00)	0.008*
Outcome, n (%)			< 0.001*
Reversion	174 (96.1%)	57 (66.3%)	
Death	7 (3.9%)	29 (33.7%)	

*, P<0.05, there were significant differences.

### Univariate and multivariate analysis

Univariate and multivariate analyses were conducted using the R package, stats (V4.2.1), to analyze the risk factors for myocardial injury caused by COVID-19.

### Identification of DEGs and GSEA

DEGs were identified in the GSE150392 and GSE169241 datasets using the R package, DESeq2. Volcano plots were drawn and visualized using the R package, ggplot2 (V3.3.3). Significant DEGs were screened with the following criteria: logFC>1.0 and *P*<0.05.

The R package, clusterProfiler package (V3.14.3), was used for GSEA. Species: Homo sapiens. The reference gene set was c2.cp.v7.2. symbols.gmt (curated) and FDR <0.25 with *P*< 0.05 were the significance cut-off criteria.

Genes in the common dysregulation pathway were obtained from the GSEA website (http://software.broadinstitute.org/gsea) and those common between GSE150392 and GSE169241 were obtained.

The R package, ggplot2 (V3.3.3), was used to generate Venn diagrams to determine the overlapping genes.

### Enrichment analysis for overlapping genes

The R package, clusterProfiler (V3.14.3), was used for Gene Ontology (GO) and Kyoto Encyclopedia of Genes and Genomes (KEGG) pathway enrichment analyses.

Overlapping genes were input into the STRING database (https://cn.string-db.org/) to construct the protein-protein interaction (PPI) network. The study organism was limited to “Homo sapiens,” while other parameters were the default. The TSV files of the PPI network were downloaded and imported into Cytoscape software (V3.7.2). The MCODE plug-in was used to search clustered subnetworks.

### Expressional difference verification and immune cells analysis

Gene expression profiles were downloaded from GSE177477; boxplots were constructed using the ggplot2 package, and the Kruskal-Wallis test was used to compare gene expression differences among uninfected healthy individuals, asymptomatic COVID-19 patients, and symptomatic COVID-19 patients with myocardial injury. A heatmap was generated using the ComplexHeatmap package to show differential expression. The cluster tree was drawn according to the gene expression of different samples using the ggplot2 package (V3.3.3) and gdendro package (V0.1.2) and was divided into two categories according to the Chebyshev distance. The CIBERSORTx website (https://cibersortx.stanford.edu/runcibersortx.php) was used to estimate the circulating immune cell types. A table containing the gene expression profile was uploaded to the CIBERSORTx website to estimate the composition of circulating immune cell types in blood samples. Significance criteria: NS, *P*≥ 0.05; *, *P*< 0.05; **, *P*<0.01; ***, *P*<0.001.

### Detecting hsTnI and IL6 levels in COVID-19 patients

IL6 and hsTnI levels in COVID-19 patients from our hospital were analyzed retrospectively. Patients whose detection index exceeded the upper limit of the reference value were in the H group and those within the range of the reference value were in the L group. These COVID-19 patients were divided into the hsTnI-L and hsTnI-H groups, and the levels of IL6 between these two groups were compared. We divided these patients into IL6-H and IL6-L groups and compared the hsTnI levels between these two groups. The reference value of hsTnI was 0.026 g/L and that of IL6 was 5.3 pg/L.

Peripheral blood was collected and centrifuged at 3000 g/min for 5 min to detect hsTnI and IL6 detection. hsTnI was detected using a chemiluminescence method (Abbott, USA), and IL6 was detected using a human Th1/Th2 subset detection kit (Cell Gene, China) and a flow cytometer (BD FACSCANTO II, USA).

### Lymphocyte subsets and hemoglobin A1c detection in COVID-19 patients

Based on hsTnI levels, we divided COVID-19 patients into hsTnI-L and hsTnI-H groups, and healthy physical examiners were selected as the control group. Lymphocyte subsets were compared among the three groups.

Peripheral blood samples were collected in ethylenediaminetetraacetic acid K2 (EDTA-K2) collection tubes for lymphocyte subset and hemoglobin A1c detection. Antibodies, including anti-CD3-PC5, anti-CD4-RD1, anti-CD8-ECD, anti-CD45-FITC, anti-CD56-RD1, and anti-CD19-ECD, were purchased from Beckman Coulter (USA). CD3^+^CD4^+^ cells represent T helper cells, CD3+CD8+ cells represent T suppressor cells, and CD4^+^CD25^+^CD127^Dim^ cells represent T regulatory cells, respectively. Lymphocyte subsets were detected using a flow cytometer (FC500MCL; Beckman Coulter, USA). Hemoglobin A1c was detected using a glycated hemoglobin analyzer (Premier Hb9210, USA).

### Detection of fasting blood glucose, triglycerides, and total cholesterol levels

The patient remained on an empty stomach for 8–12 hours, and 3 ml of fasting blood was collected and centrifuged at 3000 g/min for 5 min. The separated serum was used to detect blood glucose, triglycerides, and total cholesterol levels using a fully automated biochemical analyzer (Abbott C16000, USA). Reference values were as follows: fasting blood glucose: 3.89–6.11 mmol/l; triglycerides: 0.40–1.80 mmol/l, and total cholesterol: 3.10–5.18 mmol/l.

### Statistical analysis

All analytical methods were implemented using R packages (Foundation for Statistical Computing 2020) version 4.2.1. P *<*0.05 was considered statistically significant. If the numerical variables conformed to a normal distribution and met the variance homogeneity test criteria, a t-test was used for comparison between two groups, and a one-way ANOVA was used for comparison among three groups. If the numerical variable satisfied the normal distribution but did not satisfy the variance homogeneity test, Welch’s t-test was used for comparison between the two groups, and Welch’s one-way way ANOVA was used for comparison among the three groups. If the classification variables met the condition in which the theoretical frequency was >5 and the total sample size was >=40, the chi-square test was used for inter-group comparison; if the data met the condition of 5 >theoretical frequency >=1 and total sample size >=40, the continuous correction chi-square test (Yates’ correction) was used for inter-group comparison; if the theoretical frequency of the data was <1 or the total sample size was <40, Fisher’s exact test was used for inter-group comparison.

## Results

### Data preprocessing

**Figure 1:**
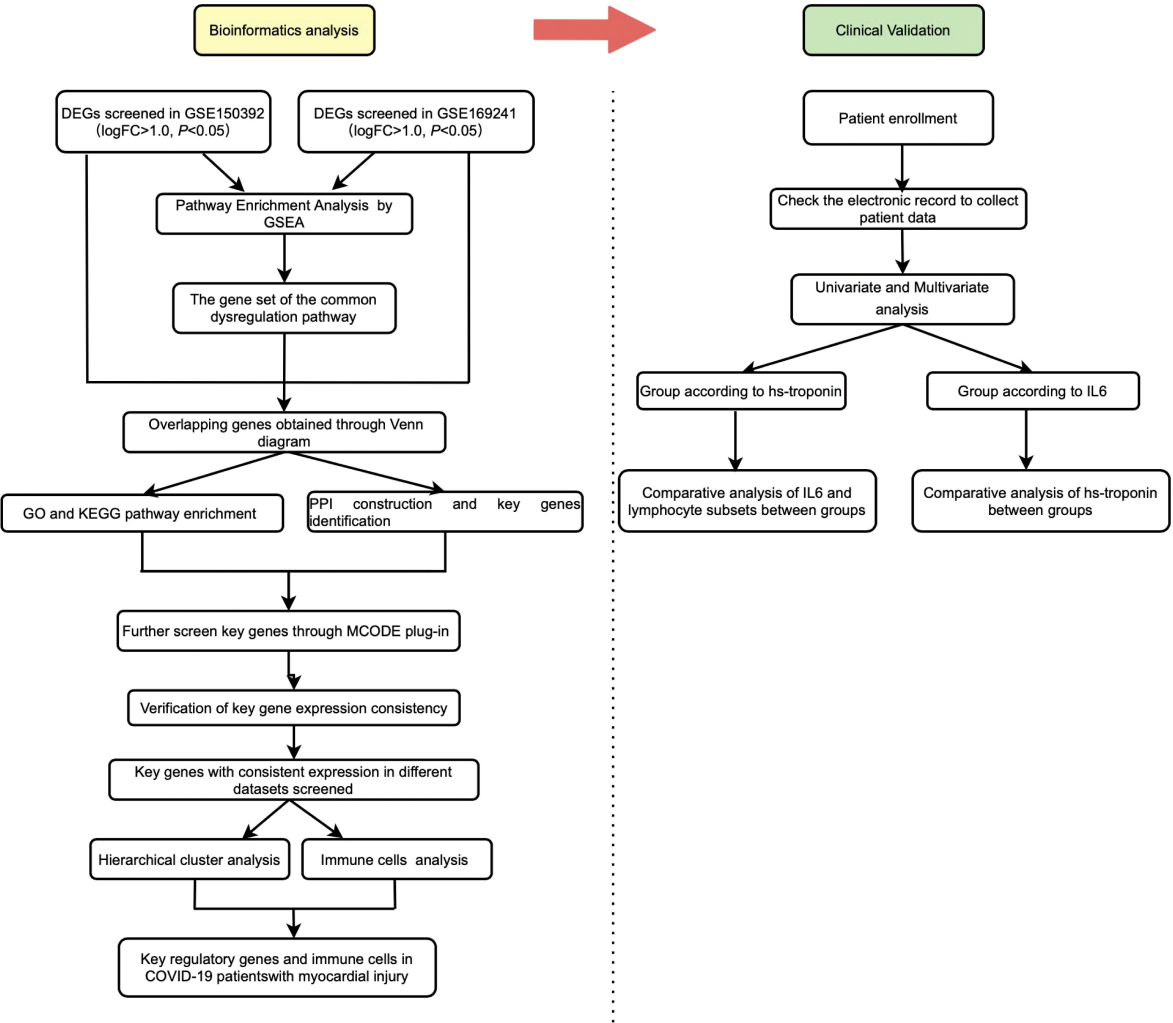
The flowchart of the study.

A detailed flowchart is shown in **Figure 1**. The GSE150392 and GSE169241 datasets were downloaded to screen DEGs in COVID-19 patients with myocardial injury. Through GSEA, we found that the commonly disordered pathway involving DEGs was the interleukin signaling pathway and GPCR ligand binding pathway. Genes involved in these two pathways were subjected to intersection analysis with DEGs to obtain key regulatory genes. The ability of these regulatory genes to identify myocardial injury was analyzed using hierarchical cluster analysis. Patients in GSE177477 were divided into two groups according to these key genes, and immune cells analysis was performed between these two groups. Finally, we analyzed clinical sample data to verify the bioinformatics results.

### Pathway enrichment analysis in COVID-19 patients with myocardial injury

**Figure 2:**
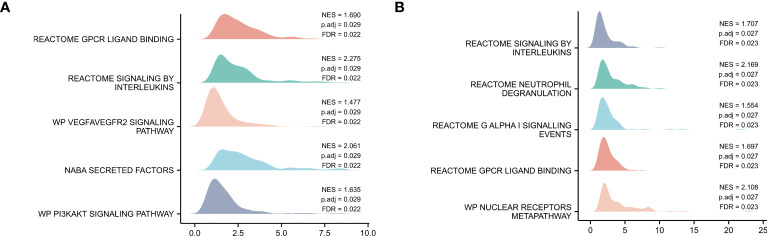
Pathway Enrichment Analysis. **(A)** GSEA mountain map of GSE150392 dataset. **(B)** GSEA mountain map of GSE169241 dataset.

GSEA of the GSE150392 and GSE169241 datasets, REACTOME SIGNALING BY INTERLEUKINS, and REACTOME GPCR LIGAND BINDING were confirmed as commonly dysregulated pathways of myocardial injury in COVID-19 (**Figure 2**).

### GO and KEGG pathway enrichment analyses

**Figure 3:**
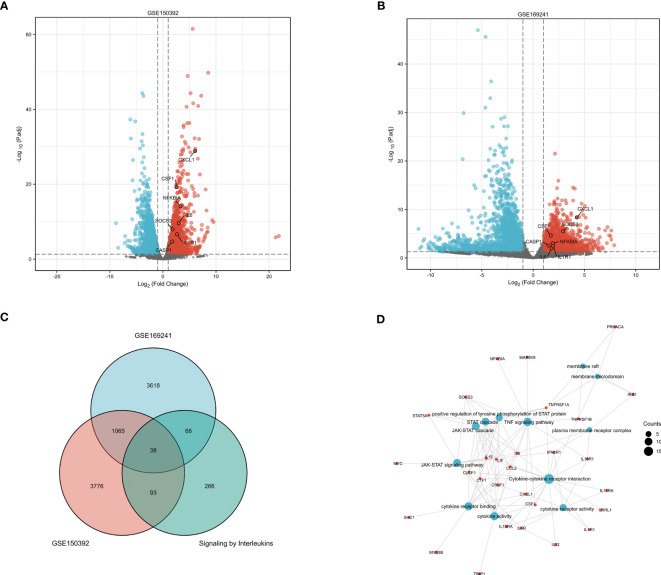
GO and KEGG Pathway Enrichment Analysis of Interleukin Pathway Related Genes **(A)** The volcano plot of DEGs in the GSE150392 dataset. **(B)** The volcano plot of DEGs in the GSE169241 dataset. **(C)** The Venn diagram of the GSE150392 DEGs, GSE169241 DEGs and gene set of interleukins signaling pathway. **(D)** GO and KEGG pathway enrichment analysis of these 38 overlapping genes.

The DEGs of the GSE150392 (**Figure 3A**) and GSE169241 datasets (**Figure 3B**) were intersected with the gene set on the interleukin signal pathway using a Venn diagram, and 38 overlapping genes were obtained (**Figure 3C**). GO and KEGG pathway enrichment analyses were performed for the 38 overlapping genes to analyze their biological functions. In terms of biological processes (BP), these 38 overlapping genes were mainly enriched in the JAK-STAT cascade, STAT cascade, and positive regulation of tyrosine phosphorylation of STAT proteins. Cell component (CC) terms were mainly enriched in the plasma membrane receptor complex, membrane rafts, and membrane microdomains. Molecular function (MF) terms were mainly concentrated in cytokine activity, cytokine receptor binding, and cytokine-cytokine receptor interaction. In the KEGG pathway analysis, cytokine-cytokine receptor interactions, TNF signaling pathway, and JAK-STAT signaling pathway were mainly enriched (**Figure 3D**).

**Figure 4:**
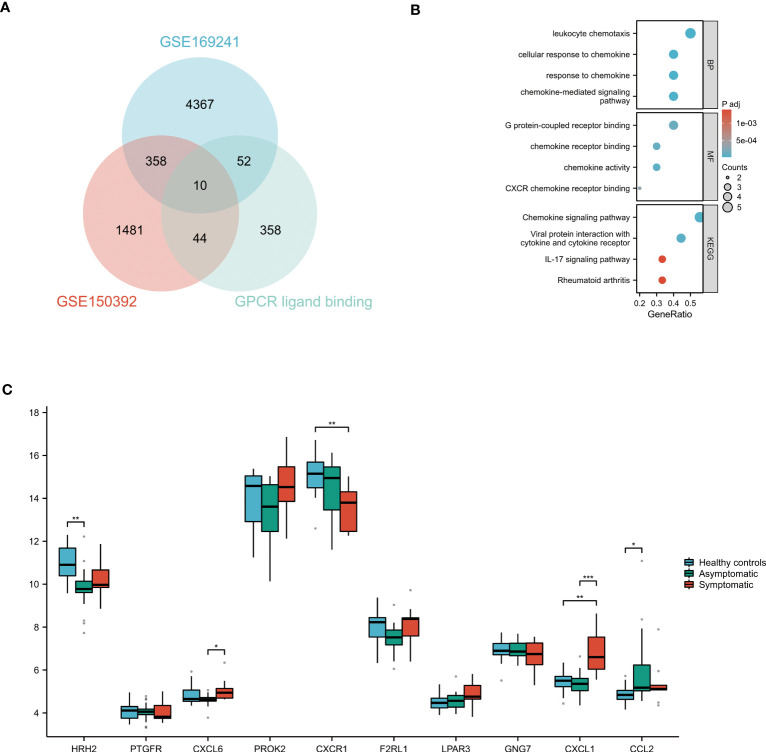
GO and KEGG Pathway Enrichment Analysis of GPCR ligand binding pathway Related Genes **(A)** The Venn diagram of the GSE150392 DEGs, GSE169241 DEGs and gene set of GPCR ligand binding pathway. **(B)** GO and KEGG pathway enrichment analysis of these 10 overlapping genes. **(C)** Validation of the key genes on the GPCR ligand binding pathway leading to myocardial injury. *P < 0.05; **P < 0.01; ***P < 0.001.

The DEGs of the GSE150392 and GSE169241 datasets were intersected with the gene set on the GPCR ligand binding pathway using a Venn diagram, and 10 overlapping genes were obtained (**Figure 4A**). GO and KEGG pathway enrichment analyses were performed for the 10 overlapping genes to analyze their biological functions. In terms of BP, these 10 overlapping genes were mainly enriched in leukocyte chemotaxis, cellular response to chemokine, response to chemokine and chemokine-mediated signaling pathway. CC terms were mainly enriched in G protein-coupled receptor binding, chemokine receptor binding, chemokine activity and CXCR chemokine receptor binding. MF terms were mainly concentrated in chemokine signaling pathway, viral protein interaction with cytokine and cytokine receptor, IL-17 signaling pathway and rheumatoid arthritis (**Figure 4B**).

### PPI network construction and identification of key genes on the interleukin signal pathway

**Figure 5:**
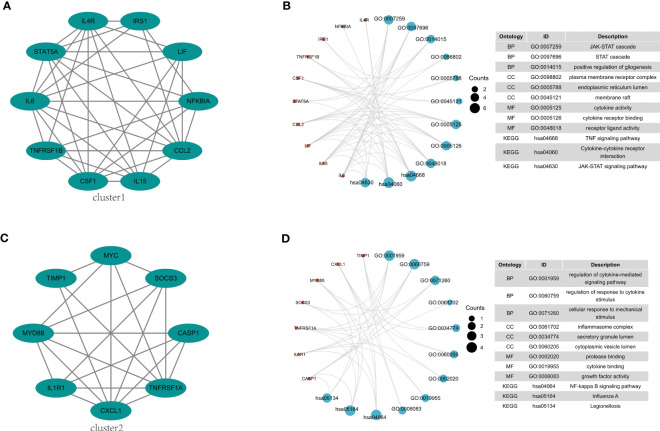
PPI network construction and pathway enrichment analysis. **(A)** Cluster 1 related genes. **(B)** GO and KEGG pathway enrichment analysis of cluster 1. **(C)** Cluster 2 related genes. **(D)** GO and KEGG pathway enrichment analysis of cluster 2.

The 38 overlapping genes were entered into the STRING database to construct a PPI network. The PPI networks were partitioned into tightly linked clusters to screen for key genes using MCODE, and two clusters were obtained. Cluster 1 contained ten genes (IL-6, STAT5A, IL4R, IRS1, LIF, NFKBIA, CCL2, IL15, CSF1, and TNFRSF1B) (**Figure 5A**), whereas cluster 2 contained eight genes (CXCL1, IL1R1, MYD88, TIMP1, MYC, SOCS3, CASP1, and TNFRSF1A) (**Figure 5C**). The KEGG pathway enrichment analysis of Cluster 1 showed enrichment of the TNF signaling pathway, cytokine-cytokine receptor interaction, and JAK-STAT signaling pathway (**Figure 5B**), while in Cluster 2 NF-κB signaling pathway, Influenza A, and Legionellosis were enriched (**Figure 5D**).

### Validation of key genes on the interleukin signal pathway in uninfected healthy individuals, asymptomatic COVID-19 cases, and symptomatic COVID-19 cases with myocardial injury

**Figure 6:**
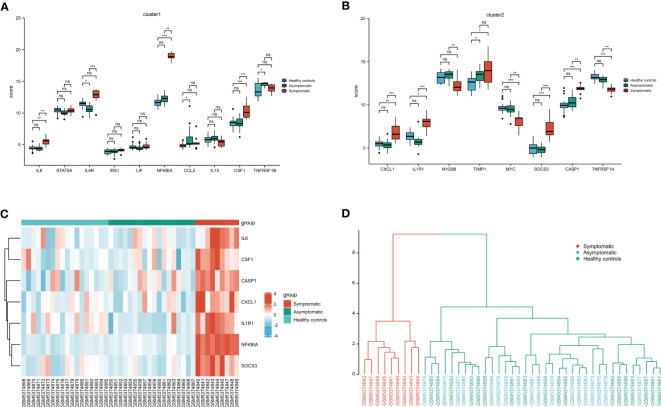
Validation of the key genes on the interleukin pathway leading to myocardial injury. **(A)** Cluster 1 related genes expression in GSE177477. **(B)** Cluster 2 related genes expression in GSE177477. **(C)** The heatmap of 7 key genes in 45 clinical samples from GSE177477. **(D)** A cluster graph of these 7 key genes including 45 clinical samples from GSE177477. NS, *P*≥ 0.05; *, *P*< 0.05; **, *P*<0.01; ***, *P*<0.001.

Clusters 1 and 2-related genes were imported into the GSE177477 dataset to further verify expression consistency. The box diagram shows that the expression levels of IL6, NFKBIA, CSF1, CXCL1, IL1R1, SOCS3, and CASP1 in symptomatic COVID-19 cases with myocardial injury were significantly higher than those in uninfected healthy individuals and asymptomatic COVID-19 cases, whereas MYC and TNFRSF1A were lower than those in uninfected healthy individuals and asymptomatic COVID-19 cases (*P*<0.05) (**Figures 6A, B**). These nine genes were verified in the GSE150392 and GSE169241 datasets, and seven genes with consistent expression were identified as key genes in the interleukin signaling pathway leading to myocardial injury (**Table 2**). Considering the inconsistent expression of MYC and TNFRSF1A in the three datasets, we selected the remaining seven genes with consistent expression to perform hierarchical clustering analysis in GSE177477. The expression of these seven genes was analyzed in GSE177477, and the results showed that IL6, NFKBIA, CSF1, CXCL1, IL1R1, SOCS3, and CASP1 were highly expressed in COVID-19 patients with myocardial injury (**Figure 6C**). These seven genes were hierarchically clustered to generate a cluster graph with 45 samples from the GSE177477 dataset. At a height of 12, the hierarchical cluster graph could be divided into two categories; one category included nine symptomatic COVID-19 patients with myocardial injury and the other category included 18 asymptomatic COVID-19 patients and 18 uninfected healthy individuals (**Figure 6D**).

**Table 2:** The expression of 9 key genes in the GSE150392 and GSE169241.

Gene Symbol	GSE	logFC	Changes	P-value	Adj. P-value
IL-6	GSE150392	2.98	UP	0.00	0.00
	GSE169241	1.80	UP	0.00	0.01
NFKBIA	GSE150392	3.32	UP	0.00	0.00
	GSE169241	1.92	UP	0.00	0.00
CSF1	GSE150392	2.54	UP	0.00	0.00
	GSE169241	1.72	UP	0.00	0.00
CXCL1	GSE150392	6.06	UP	0.00	0.00
	GSE169241	4.25	UP	0.00	0.00
IL1R1	GSE150392	2.65	UP	0.00	0.00
	GSE169241	1.95	UP	0.00	0.00
SOCS3	GSE150392	1.84	UP	0.00	0.00
	GSE169241	2.92	UP	0.00	0.00
CASP1	GSE150392	1.77	UP	0.00	0.00
	GSE169241	1.50	UP	0.00	0.00
MYC	GSE150392	1.82	UP	0.00	0.00
	GSE169241	1.58	UP	0.01	0.03
TNFRSF1A	GSE150392	1.38	UP	0.00	0.00
	GSE169241	1.08	UP	0.01	0.02

### Validation of key genes on GPCR ligand binding pathway in uninfected healthy individuals, asymptomatic COVID-19 cases, and symptomatic COVID-19 cases with myocardial injury

These 10 overlapping genes on GPCR ligand binding pathway were further validated in uninfected healthy individuals, asymptomatic COVID-19 cases, and symptomatic COVID-19 cases with myocardial injury in GSE177477. The box diagram showed that the expression levels of CXCL1 in symptomatic COVID-19 cases with myocardial injury were significantly higher than those in uninfected healthy individuals and asymptomatic COVID-19 cases (*P*<0.05) (**Figure 4C**).

### Immune cells analysis in GSE177477

**Figure 7:**
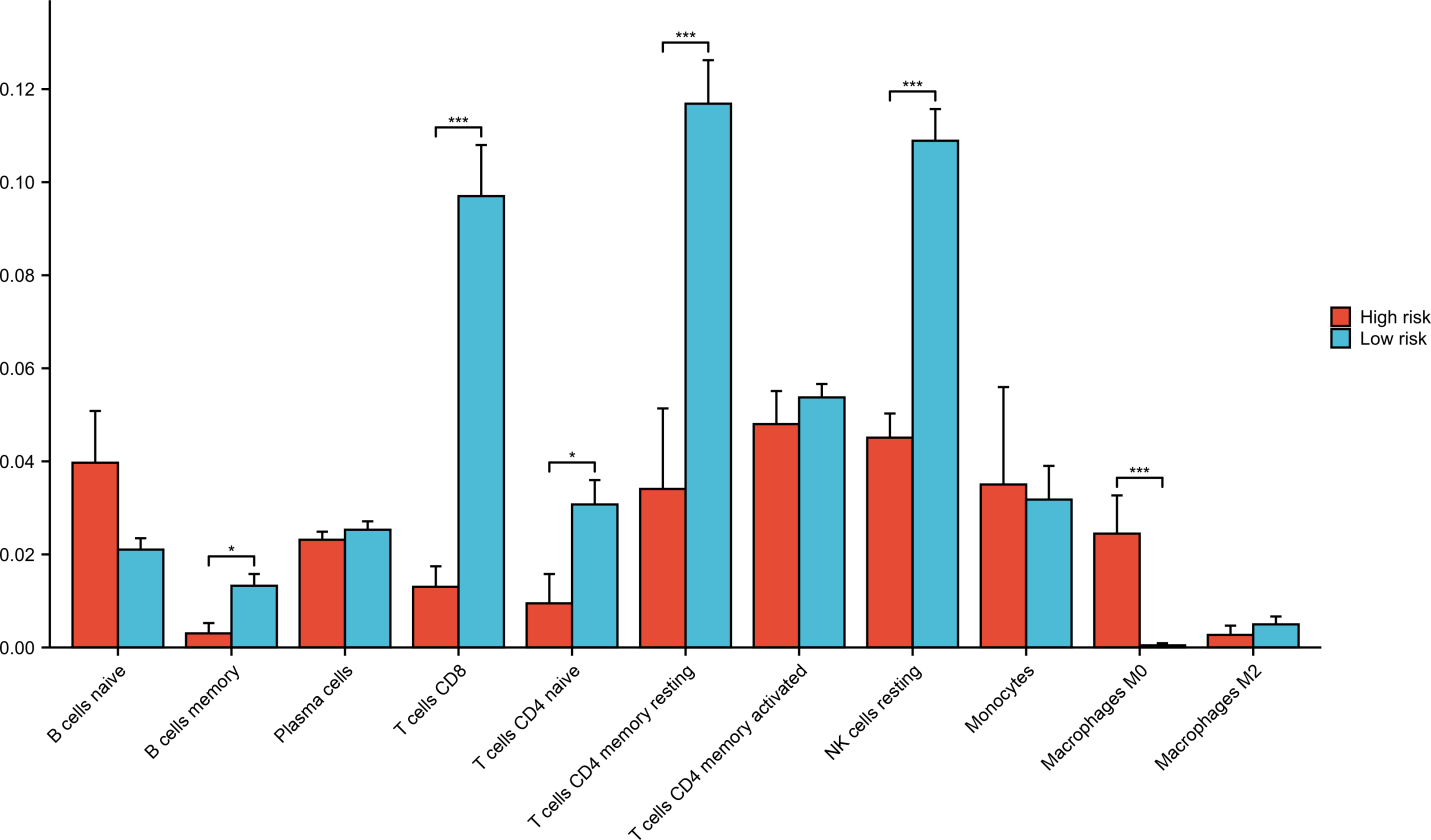
Immune cells analysis in low-risk and high-risk groups. Forty-five samples in GSE177477 were divided into high-risk group (symptomatic COVID-19 cases with myocardial injury, n=9) and low-risk group (asymptomatic COVID-19 cases and uninfected healthy individuals, n=36) according to the hierarchy. *P < 0.05; ***P < 0.001.

Forty-five samples in GSE177477 were divided into high-risk (symptomatic COVID-19 patients with myocardial injury, n = 9) and low-risk (asymptomatic COVID-19 patients and uninfected healthy individuals, n = 36) groups according to the hierarchy. The proportions of immune cells in the high- and low-risk groups was analyzed. The proportions of B memory cells, CD8^+^ T cells, CD4^+^ naïve T cells, CD4^+^ memory resting T cells, and NK resting cells in the high-risk group were significantly lower than those in the low-risk group, whereas that of the M0 Macrophages in the high-risk group was significantly higher than those in the low-risk group (*P*<0.05) (**Figure 7**).

### Regression analysis of clinical samples

**Figure 8:**
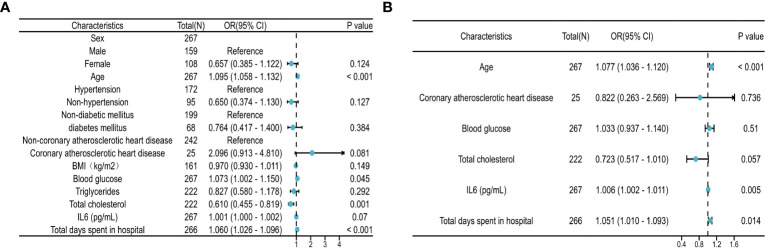
Regression analysis of risk factors. **(A)** Forest plot of univariate regression analysis. **(B)** Forest plot of multivariate regression analysis. The regression analysis of risk factors based on 267 clinical samples. HR>1 was considered a risk factor for myocardial injury, while HR<1 was considered a protective factor. The P threshold in univariate regression analysis was 0.1.

Univariate and multivariate analyses were conducted for various clinical factors (**Figure 8**). To avoid interference from confounding factors, we increased the looseness of the *P*-value in the univariate analysis. If the *P* value was less than 0.1 in univariate analysis, the factor was included in the multivariate analysis. Univariate analysis revealed that age, fasting blood glucose level, total cholesterol level, and length of hospital stay may be factors influencing myocardial injury caused by COVID-19. Multivariate analysis showed that age, IL6, and hospital stay were factors influencing myocardial injury caused by COVID-19.

### The levels of IL6 and hsTnI in COVID-19 patients

**Figure 9:**
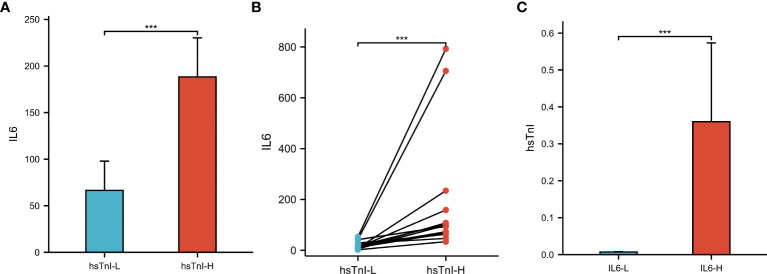
The levels of IL6 and hsTnI in COVID-19 patients. **(A)** Non-pair comparative analysis of IL6 between hsTnI-L and hsTnI-H groups. **(B)** Paired comparative analysis of IL6 between hsTnI-L and hsTnI-H groups. **(C)** Non-pair comparative analysis of hsTnI between IL6-L and IL6-H groups. The hsTnI-H group included 86 patients, while the hsTnI-L group included 181 patients. The IL6-H group included 25 patients, while the IL6-L group included 242 patients. The normal distribution between groups was not met (*P*<0.05), so the nonparametric test was selected. ***P < 0.001.

Based on hsTnI levels, COVID-19 patients were divided into two groups to analyze the level of IL6. Those with levels higher than the normal reference were defined as the hsTnI-H group (n=86), and those within the reference range were defined as the hsTnI-L group (n=181). The level of IL6 in the hsTnI-H group was 39.57 (13.75, 114.16) (pg/mL), and that in the hsTnI-L group was 10.14 (4.62, 30.84) (pg/mL). The IL6 expression in the hsTnI-H group was significantly higher than that in the hsTnI-L group (*P*<0.05; **Figure 9A**). Follow-up of the 16 COVID-19 patients showed that with an increase in hsTnI, IL6 expression increased significantly (*P*<0.05) (**Figure 9B**).

According to IL6 expression, patients with COVID-19 were divided into two groups to analyze hsTnI levels. Those with levels higher than the normal reference were defined as the IL6-H group (n=25), and those within the reference range were defined as the IL6-L group (n=242). The level of hsTnI in the IL6-H group was 0.02 (0.01, 0.05) (ug/L), and that in the IL6-L group was 0.01 (0.00,0.01) (ug/L). The hsTnI levels in the IL6-H group were significantly higher than those in the IL6-L group (*P*<0.05; **Figure 9C**).

**Figure 10:**
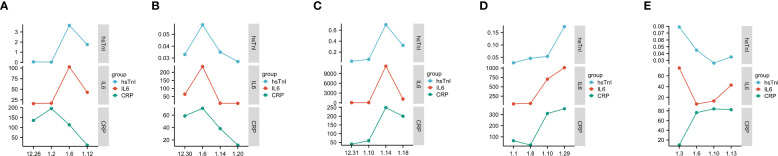
Trends in IL6, CRP, and hsTnI. **(A–E)** Trends in CRP, IL6, and hsTnI in five different patients. The X-axis represents the date, while the Y-axis represents the CRP, IL6, and hsTnI values. CRP unit is mg/L, IL6 unit is pg/L, hsTnI unit is ug/L.

The trend in IL6 and hsTnI levels in COVID-19 patients with myocardial injury was further analyzed, and the results showed that the changes in IL6 and hsTnI levels were strikingly similar. Considering that both CRP and IL6 are inflammatory indicators, we also analyzed the changing trend of CRP and hsTnI, and the correlation between IL6 and hsTnI levels was better than that between CRP and hsTnI (**Figure 10**).

### Lymphocyte subsets in COVID-19 patients

**Figure 11:**
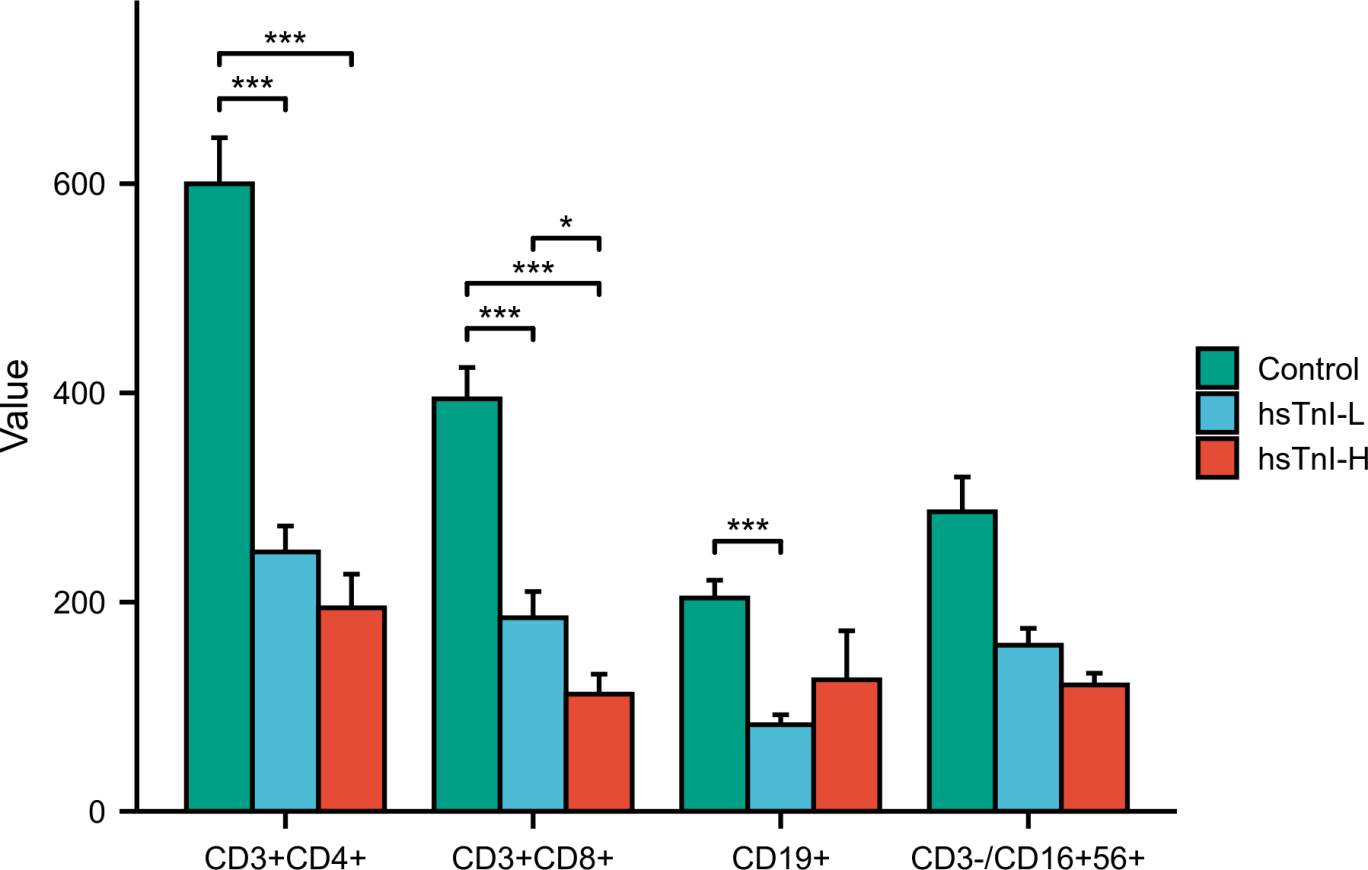
Comparative analysis of lymphocyte subsets among hsTnI-L, hsTnI-H and control groups. The normal distribution between groups was not met (*P*<0.05), so the nonparametric test was selected. The homogeneity test of variance showed that the variances of each group were not equal (*P*<0.05), and the correction method was selected. *P < 0.05; ***P < 0.001.

Among the 267 enrolled COVID-19 patients, lymphocyte subsets were simultaneously detected in 104 patients. Based on hsTnI levels, these 104 patients were divided into the hsTnI-L group (n=59) and hsTnI-H group (n=45). Healthy physical examiners without myocardial injury (n=41) were selected as the control group. The lymphocyte subsets were compared among the three groups. Compared with the control group, the counts of CD3^+^CD4^+^ T cells, CD3^+^CD8^+^ T cells, and CD19^+^ B cells decreased in the hsTnI-L group, and those of CD3^+^CD4^+^ T cells and CD3^+^CD8^+^ T cells decreased in the hsTnI-H group (*P*<0.05). Compared to the hsTnI-L group, the count of CD3^+^CD8^+^ T cells decreased in the hsTnI-H group (*P*<0.05) (**Figure 11**).

## Discussion

SARS-COV-2 infection not only causes lung injury but also myocardial injury. As a serious complication of COVID-19, myocardial injury can lead to high mortality (14). Our clinical data showed that COVID-19 patients combined with myocardial injury had poor outcomes and the mortality rate was significantly higher than those without myocardial injury (*P*<0.05). Acute myocardial injury caused by COVID-19 is associated with adverse outcomes, including male sex, old age, and cardiovascular comorbidities (15). Based on the clinical features of our patients, there were differences in age, BMI, total cholesterol, IL6, and adverse outcomes between COVID-19 patients with and without myocardial injury. The host mechanisms of myocardial injury associated with COVID-19 include tissue damage caused by viral infection and replication, host immune disorders, and cytokine storms (16). Univariate and multivariate regression analyses revealed that age, IL6 levels, and hospital stay were risk factors for myocardial injury (*P*<0.05). Within the normal cholesterol range, higher cholesterol levels appeared to be a protective factor (P=0.057). We analyzed the clinical data of 267 patients and found that the total cholesterol level in the hsTnI-L was higher than that in the hsTnI-H group (*P*<0.05). Zhang et al. found that cholesterol levels in the non-ICU/severe group were also higher than those in the ICU/severe group (P<0.05 (17). The total cholesterol levels in our patients did not exceed the upper limit of the normal range (the reference range of cholesterol was 3.10–5.18 mmol/L). The total cholesterol level in the myocardial injury group was lower than that in the non-myocardial injury group, which may be due to cholesterol reduction. As a precursor of corticosteroids, the reduction in cholesterol leads to a reduction in the synthesis of corticosteroids and weakens immunity and antiviral ability (18, 19). For *in vitro* experiments, hiPSC-CMs were infected with SARS-COV-2 directly (10). Biopsies of the myocardium from COVID-19 patients with myocarditis showed that SARS-COV-2 infected myocardial cells caused myocardial fiber damage (20, 21). T lymphocytes are activated, and virus-specific cytotoxic T lymphocytes cause hemolysis of the infected myocardium, leading to serious myocardial damage (22). Although many studies have examined the mechanism of myocardial injury caused by COVID-19, the detailed mechanism remains unclear owing to the complex network of cardiac and immune cell types involved. Therefore, it is helpful to prevent and treat myocardial injuries in COVID-19 patients by studying the detailed mechanisms underlying COVID-19 and identifying key regulatory molecules for timely intervention.

By conducting GSEA for the GSE150392 and GSE169241 datasets, the interleukin signaling pathway and GPCR LIGAND BINDING pathway were confirmed to be the common disordered pathways of myocardial injury related to COVID-19. By verifying the key genes on these two pathways, seven genes on the interleukin pathway and one genes (CXCL1) on the GPCR LIGAND BINDING pathway were confirmed to be related to the disease. CXCL1 was the common gene of the interleukin pathway and GPCR ligand binding pathway. Therefore, we inferred that the interleukin pathway was the main pathway of myocardial injury caused by COVID-19. The interleukin signaling pathway includes multiple molecules, such as IL1, IL2, IL3, IL4, IL6, IL7, IL10, IL13, IL17, IL20, and IL22. A bioinformatics study based on the transcriptomics of SARS-COV-2 infected myocardium showed that the pathological mechanism of myocardial injury was mainly concentrated in inflammatory signaling pathways (5). The high inflammatory burden is the main cause of myocardial injury associated with COVID-19 (23). High levels of IL17A and up-regulated JAK2-STAT3 signaling pathway can promote inflammatory reactions, induce cardiomyocyte apoptosis, and ultimately aggravate myocardial ischemia and reperfusion (I/R) injury (24). Inhibition of inflammatory factors can help alleviate the progression of myocardial injury. IL1 receptor antagonist (Anakinra) can competitively inhibit the binding of IL1α/IL1β and IL1 type I receptors and neutralize the activity of key mediators of autoimmune inflammation and immune process (25). Tocilizumab is an IL6 receptor blocker that effectively blocks the IL6 signal transduction pathway and is an effective therapeutic drug for COVID-19 patients. Tocilizumab can reduce the intubation risk, mortality, and hospital stay in COVID-19 patients without increasing the risk of superimposed infections (26). Nifuroxazide improves myocardial oxidation and attenuates LPS-induced myocardial injury by interrupting TLR4/NALPR3/IL-1β signaling (27). The interleukin signaling pathway plays a key role in myocardial injury in COVID-19 patients. To further explore whether multiple pathways were involved in myocardial injury, we performed an intersection analysis for DEGs of GSE150392 and GSE169241 with key genes in the interleukin signaling pathway and obtained 38 overlapping genes. Through GO and KEGG enrichment analyses on these 38 overlapping genes, we concluded that cytokine-cytokine receptor interaction, TNF signal pathway, JAK-STAT signal pathway, and NF-κB signaling pathways may participate in myocardial injury caused by SARS-COV-2 infection together. NF-κB signaling pathway, as a key regulatory factor of inflammation, affected the inflammatory state of COVID-19 patients (28). TNF and other inflammatory factors can also activate host immune cells through the NF-κB signaling pathway (29). In addition, IL6 participates in inflammatory reactions through the IL6-STAT3 signal pathway (30). Therefore, myocardial injury associated with COVID-19 is caused by multiple inflammation-related pathways, among which the interleukin signaling pathway may be the key inflammatory pathway.

To identify key regulatory molecules in the inflammatory pathway, we constructed a PPI network based on 38 overlapping genes and obtained two clusters. Cluster 1 contained 10 genes that were mainly concentrated in the TNF signaling pathway, cytokine-cytokine receptor interaction, and the JAK-STAT signaling pathway. Cluster 2 contained 8 genes that were concentrated in the NF-κB signaling pathway, Influenza A, and Legionellosis. Eighteen key genes in these two clusters were further verified in GSE177477, GSE150392, and GSE169241; seven genes (IL6, NFKBIA, CSF1, CXCL1, IL1R1, SOCS3, and CASP1) were consistently expressed in these three datasets. Hierarchical clustering of these seven genes showed that at a height of 12 m, COVID-19 patients with myocardial injury could be distinguished from those with COVID-19 alone and uninfected healthy individuals. The risk of myocardial injury caused by COVID-19 could be predicted by constructing a model containing these seven genes.

Samples in GSE177477 were divided into high- and low-risk groups based on the presence of myocardial injury. Compared to the low-risk group, M0 macrophages were increased in the high-risk group, while B memory cells, CD8^+^ T cells, CD4^+^ naïve T cells, CD4^+^ memory resting T cells, and NK resting cells significantly decreased (*P*<0.05). SARS-COV-2 invaded multiple organs and tissues, leading to serious damage to the respiratory and immune systems (31). When SARS-COV-2 invades the immune system involving the lymph nodes, tonsils, spleen, and bone marrow, lymph node atrophy, T lymphocytes, and B lymphocytes decrease significantly, especially T lymphocytes (32). In a study by Lian et al., M0 macrophages in COVID-19 were significantly increased, while the levels of M2 macrophages were not significantly altered. Our results are consistent with the conclusions of Lian et al., mainly because SARS-COV-2 infection tended to trigger a cascade of inflammatory pathways in M0 and M1 macrophages but not in M2 macrophages (33). To further verify whether there was an imbalance in immune cells in COVID-19 patients, we analyzed the lymphocyte subsets of COVID-19 patients in our hospital. Compared with the healthy control group, the counts of CD3^+^CD4^+^ and CD3^+^CD8^+^ T cells in COVID-19 patients decreased significantly. In particular, the numbers of CD3^+^CD4^+^ and CD3^+^CD8^+^ T cells decreased to a greater extent in COVID-19 patients with myocardial injury. Zhang et al. reported that CD4^+^ and CD8^+^T cells in patients with severe COVID-19 decreased significantly, which is consistent with our results (34). The lower the T-lymphocyte count, the higher the incidence of composite endpoint events. SARS-COV-2 infection causes T-cell depletion and affects cellular immunity (35). CD8^+^T cells are key determinants of prognosis during acute COVID-19 infection (36). We found that the number of CD8^+^T cells in COVID-19 patients with myocardial injury was significantly lower than those in COVID-19 patients without myocardial injury (*P*<0.05). Detection of lymphocyte subsets can help clinicians predict the risk of poor prognosis in patients. In our study, the number of B cells in COVID-19 patients was lower than that in healthy controls; however, the number of B cells in COVID-19 patients with myocardial injury was higher than that in patients without myocardial injury, although the difference was not statistically significant. Jimenez pointed out that the percentage of B cells in COVID-19 patients increased as the severity of the disease increased (37). B-cells are closely associated with humoral immunity and may lead to host cell injury. We speculate that myocardial injury may be caused by excessive activation of humoral immunity.

As a pro-inflammatory cytokine, IL6 controls the proliferation, survival, and orientation of T cells and can be used as an inflammatory marker for severe COVID-19 infection. In our study, IL6 was highly expressed in symptomatic COVID-19 patients with myocardial injury. IL6 participated in multiple inflammation-related pathways such as the cytokine receptor interaction, TNF signaling pathway, JAK-STAT signaling pathway, NF-κB signaling pathway, etc. We analyzed IL6 and hsTnI levels simultaneously in 267 patients with COVID-19 and found that the level of IL6 in COVID-19 patients with myocardial injury was higher than that in patients without myocardial injury. Similarly, the IL6-H group had higher hsTnI than the IL6-L group. The trends of IL6, CRP, and hs-TnI were analyzed in COVID-19 patients with myocardial injury. The results showed that the trend of IL6 and TnI were very similar; however, the trends of CRP and TnI were not identical. Thus, we inferred that IL6 may play an important role in myocardial injury. IL6 is a pleiotropic cytokine and a high level of IL6 can induce inflammatory reactions. Myocardial injury is mostly attributed to reperfusion injury and inflammatory reaction (38). The aberration in IL6 level was associated with COVID-19 complications, including neurological and cardiovascular diseases (39, 40). The increase in serum IL6 level is related to severe COVID-19 and is a good biomarker for distinguishing between severe and mild cases. Tocilizumab, a monoclonal antibody against IL6 is an effective drug for patients with severe COVID-19 (41). Drugs that inhibit IL6 levels also reduce inflammation, minimize complications, and reduce mortality rates. IL6 plays a role in the pathogenesis of SARS-COV-2 infection-related myocardial injury. Therefore, it is important to monitor the level of IL6 during disease treatment.

However, there are some limitations to our study. First, the datasets used to verify genes related to the interleukin pathway were obtained only from the GEO database, and there were insufficient clinical samples to verify the expression of these genes. Second, GSE177477, as a validation set, contained nine COVID-19 patients with myocardial injury; however, we could not directly distinguish whether the myocardial injury was caused by COVID-19. Finally, the number of samples used in the validation set was insufficient.

With the constant variation in COVID-19 cases, the transmission capacity was stronger. Although its pathogenicity has declined, there are many severe diseases owing to the large global population. Myocardial injury is a serious complication of COVID-19 that is associated with high mortality rates. Using bioinformatics, we found abnormal interleukin pathways in COVID-19 patients with myocardial injury, and the proportions of T cells, B cells, and NK cells in these patients were reduced.

Through clinical sample verification, the level of IL6 in COVID-19 patients with myocardial injury increased, whereas lymphocyte subsets decreased. These results were consistent with our bioinformatics results. Inhibition of the expression of IL6 and monitoring of lymphocyte homeostasis may help prevent myocardial injury in COVID-19 patients.

## Conclusions

Through bioinformatics analysis, we concluded that the interleukin signaling pathway is the main mechanism implicated in myocardial injury associated with COVID-19. The myocardial injury caused by COVID-19 was associated with multiple inflammatory pathways, and IL6, NFKBIA, CSF1, CXCL1, IL1R1, SOCS3, and CASP1 were key genes of the inflammatory pathway. Myocardial injury in COVID-19 patients is closely related to adverse outcomes. Age, IL6 and length of hospital stay may be factors influencing myocardial injury caused by COVID-19. In COVID-19 patients with myocardial injury, the level of IL6 increased while the lymphocyte count decreased. IL6 may participate in myocardial injury through the interleukin signaling pathway in COVID-19 patients.

## Data availability statement

The original contributions presented in the study are included in the article/supplementary material. Further inquiries can be directed to the corresponding authors.

## Ethics statement

The studies involving human participants were reviewed and approved by Ethics Committee of the First Affiliated Hospital of Zhejiang University of Traditional Chinese Medicine. Written informed consent for participation was not required for this study in accordance with the national legislation and the institutional requirements. Written informed consent was not obtained from the individual(s) for the publication of any potentially identifiable images or data included in this article.

## Author contributions

HC was responsible for checking the patient’s electronic medical record. LZ carried out clinical data statistical analysis and drawing. YY and PC were responsible for bioinformatics analysis. TC was a major contributor in writing the manuscript. WM was the main designer of the experiment. All authors contributed to the article and approved the submitted version.
